# Effective Neutralizing Antibody Response Against SARS-CoV-2 Virus and Its Omicron BA.1 Variant in Fully Vaccinated Hematological Patients

**DOI:** 10.1007/s10238-023-01223-w

**Published:** 2023-10-28

**Authors:** Danilo De Novellis, Veronica Folliero, Valentina Giudice, Luca Pezzullo, Giuseppina Sanna, Raffaele Fontana, Roberto Guariglia, Carla Zannella, Laura Mettivier, Idalucia Ferrara, Giovanni Boccia, Maria Teresa Buonanno, Maria Carmen Martorelli, Serena Luponio, Andrea Crudele, Pasquale Pagliano, Anna Maria Sessa, Francesca Velino, Maddalena Langella, Aldo Manzin, Massimiliano Galdiero, Carmine Selleri, Gianluigi Franci, Bianca Serio

**Affiliations:** 1 Department of Medicine, Surgery, and Dentistry, University of Salerno, Baronissi, Italy; 2 Hematology and Transplant Center, University Hospital “San Giovanni di Dio e Ruggi d’Aragona”, Salerno, Italy; 3 Microbiology and Virology, University Hospital “San Giovanni di Dio e Ruggi d’Aragona”, Salerno, Italy; 4 Department of Biomedical Sciences, Microbiology and Virology Unit, University of Cagliari, Cittadella Universitaria, 09042 Monserrato, Italy; 5 Department of Experimental Medicine, University of Campania “Luigi Vanvitelli”, 80138 Naples, Italy

**Keywords:** Hematological malignancies, COVID-19, SARS-CoV-2, Neutralizing antibodies, Immunity

## Abstract

SARS-CoV-2 and its variants cause CoronaVIrus Disease 19 (COVID-19), a pandemic disease. Hematological malignancies increase susceptibility to severe COVID-19 due to immunosuppression. Anti-SARS-CoV-2 neutralizing antibodies protect against severe COVID-19. This retrospective real-life study aimed to evaluate seropositivity and neutralizing antibody rates against SARS-CoV-2 and its Omicron BA.1 variant in hematological patients. A total of 106 patients with different hematologic malignancies, who have mostly received three or more vaccine doses (73%), were included in this study. Serum was collected between May and June 2022. The primary endpoint was anti-SARS-CoV-2 antibody response against ancestral (wild type; wt) and Omicron BA.1 virus, defined as a neutralizing antibody titer ≥ 1:10. Adequate neutralizing antibody response was observed in 75 (71%) and 87 (82%) of patients for wt and Omicron BA.1 variants, respectively.

However, patients with B-cell lymphoproliferative disorders and/or those treated with anti-CD20 monoclonal antibodies in the prior 12 months showed a lower seropositivity rate compared to other patients against both Omicron BA.1 variant (73% vs 91%; *P* = 0.02) and wt virus (64% vs 78%; *P* = 0.16). Our real-life experience confirmed that full vaccination against SARS-CoV-2 induces adequate neutralizing antibody protection for both the wt virus and Omicron BA.1 variants, even in hematological frail patients. However, protective measures should be maintained in hematological patients, especially those with B-cell lymphoproliferative diseases treated with anti-CD20 monoclonal antibodies, because these subjects could have a reduced neutralizing antibody production.

## Introduction

Since the initial outbreak, several variants of the SARS-CoV-2 virus have emerged, showing differences in immune responses, infectivity, incidence of severe disease, reduction in neutralization by antibodies, and decreased response to available vaccines [1, 2]. The Omicron variant and its lineage variants are currently the most widespread and monitored variants of concern (VOCs) [3]. Current vaccines, including mRNA- or adenovirus viral vector-based formulations, have demonstrated high efficacy in protecting against SARS-CoV-2 infection and severe COVID-19 [4]. However, new VOCs and VOIs raise concerns about the efficacy of available vaccines against novel variants. Previous SARS-CoV-2 infection could elicit effective immune responses against reinfections in the majority of subjects, protecting against severe COVID-19, as well as against variant infections [5]. Several risk factors for severe COVID-19 have been identified, including age, cardiovascular and respiratory diseases, and sex [6–9]. Underlying immunosuppression could be a risk factor of severe COVID-19, as the combination of some immunosuppressive drugs, such as methotrexate plus mycophenolate, can synergistically deplete T lymphocytes and impair viral clearance, or reduce neutralizing antibody production [6–8]. Hematological malignancies are often accompanied by a deep immunosuppressive status, caused by severe neutropenia, T-/B-cell dysfunctions, and secondary hypogammaglobulinemia [10–13]. Antibody responses after anti-SARS-CoV-2 vaccines and natural infections in hematological patients have been widely evaluated in several trials showing different and conflicting results, such as in patients with chronic lymphocytic leukemia [14–18]. Specific anti-SARS-CoV-2 neutralizing antibody titers can predict vaccination efficacy and protection against severe COVID-19 [19, 20]. This single-center real-life retrospective study reports the prevalence of neutralizing antibody response against SARS-CoV-2 and its Omicron BA.1 variant in a cohort of hematological patients with various malignant disorders, and the impact of several clinical features on neutralizing antibody production during hematological diseases.

## Materials and methods

### Patients

This retrospective study included 106 consecutive subjects who were screened for hematological malignancies according to international guidelines according to WHO criteria [10, 11] at the Hematology and Transplant Center, University Hospital “San Giovanni di Dio e Ruggi d’Aragona,” Salerno, Italy. Clinical characteristics are summarized in Table 1. Almost all enrolled patients (95%; *N* = 94) received anti-SARS-CoV-2 vaccination. Serum samples were collected between May and June 2022 in accordance with the Declaration of Helsinki and protocols approved by local Ethic Committee “Campania Sud” (Brusciano, Naples, Italy; prot./SCCE n. 24988). Primary endpoint was the neutralizing anti-SARS-CoV-2 antibody response against ancestral and Omicron BA.1 variants evaluated as percentage of responders’ subjects. Inclusion criteria were: age ≥ 18 years old; previous diagnosis of hematological malignancy outside clinical trials; and previous anti-SARS-CoV-2 vaccination or viral infection. A group of healthy controls (*N* = 20; median age, 58 years; range, 19–97 years old; M/F, 11/8) from another our parallel study (unpublished data) was used for neutralizing antibody titer comparison.

**Table 1:** Patients’ characteristics

Characteristics	*N* = 106
Median age, years (range)	65 (18–98)
Male/Female, *n* (%)	65/41 (61/39)
Hematological malignancy, * n* (%)	
*Multiple myeloma*	24 (22)
*Myelodysplastic syndrome*	14 (13)
*Acute leukemias*	22 (21)
*Non-Hodgkin Lymphoma*	22 (21)
*Hodgkin Lymphoma*	7 (7)
*Chronic myeloid leukemia*	4 (4)
*Chronic Lymphocytic leukemia*	3 (3)
*Allogeneic stem cell transplant*	7 (7)
*Others*	3 (2)
Anti-SARS-CoV-2 vaccination, * n* (%)	94/106 (95)
Number of vaccine doses, * n* (%)	
1	5 (5)
2	13 (12)
3	63 (61)
4	13 (12)
Type of vaccine, * n* (%)	
*Monovalent mRNA*	82 (77)
*Mixed*	7 (7)
*Not available*	5 (5)
Prior natural SARS-CoV-2 infection, * n* (%)	36/106 (33)
Number of SARS-CoV-2 infection, * n* (%)	
1	34 (32)
2	2 (2)
Specific treatments in the last 12 months, * n* (%)	
*Anti-CD20 monoclonal antibodies*	22 (21)
*Anti-CD38 monoclonal antibodies*	14 (13)
*Azacytidine*	23 (22)
*Immunosuppressive agents*	6 (6)
*High-dose chemotherapy*	26 (25)
Time from last vaccine dose or infection, days, median (range)	135(15–504)
Time from last vaccine dose or infection, * n* (%)	
< 30 days	9 (9)
> 180 days	21 (20)

### Neutralizing antibody titer

Serum neutralization assay (SNA) was performed in the Biosafety Level 3 (BSL-3) laboratory. SNA was conducted using 96-well flat bottom microtiter plates. Serum samples were diluted (1:10; 1:40; 1:160; 1:640) in triplicates and mixed with 100 TCID_50_ of SARS-CoV-2 virus (clinical isolate, kindly donated by the Lazzaro Spallanzani Hospital of Rome, Italy), and BA.1 variant (clinical isolate, strain EPI_ISL_13398512) at 37 °C. Serum/virus mixes (100 µl) were transferred to 96-wells containing 5 × 10^5^/ml adherent Vero E6 and Vero 76 (ATCC, Manassas, Virginia, United States) cells, respectively, seeded the day before in. Plates were then incubated for three or five (BA.1) days at 37 °C in 5% CO_2_. Negative control consisted of exposing an uninfected cell monolayer to sera, while infected cells not treated with sera represented the positive control. After incubation, cytopathic effect development was detected using a crystal violet solution with 5% formaldehyde, as crystal violet only stains intact cell monolayers not destroyed by viral infection. Subsequently, monolayers were washed, and absorbance was read at 595 nm wavelength. Neutralizing power of individual dilutions was calculated by setting the mean absorbance of negative control equal to 100%. Neutralization titers were expressed as the dilution of serum that neutralized 90% of inoculated wells. Values ≥ 1:10 were considered as an adequate presence of neutralizing antibodies, in line with the recommendations provided by the European Centre for Disease Prevention and Control and supported by previously published literature [21–25].

### Statistical analysis

Data were collected in spreadsheets and analyzed using R statistical software (v. 4.0.5; RStudio) and SPSS (v. 25; IBM). Differences between groups were investigated by Chi-square, Fisher’s, Wilcoxon signed-rank, or unpaired two-tailed *t* tests. Univariate and multivariate logistic regression models were used for investigation of the impact of independent variables on outcomes. A *P *value < 0.05 was considered statistically significant.

## Results

### Clinical characteristics at enrollment

A total of 106 consecutive subjects were included in this retrospective study (Table 1), mostly diagnosed with Hodgkin or non-Hodgkin lymphomas (*N* = 7 and *N* = 22, respectively; 28%), multiple myeloma (*N* = 24; 22%), acute leukemias (*N* = 22; 21%), or myelodysplastic syndrome (*N* = 14; 13%). Treatments included anti-CD20 or anti-CD38 monoclonal antibody administration in 21% and 13% of patients (*N* = 22 and *N* = 14, respectively). Azacytidine, high-dose chemotherapy, and immunosuppression were used in 22% (*N* = 23), 25% (*N* = 26), and 6% (*N* = 6) of cases, respectively. Among patients, 94 (95%) had received previous anti-SARS-CoV-2 vaccination with most receiving three doses (61%; *N* = 63), while 24% of subjects had two or four administrations (*N* = 13 or *N* = 13, respectively), and only 5% (*N* = 5) received only one dose. In most cases (*N* = 82; 77%), patients had only monovalent mRNA-based vaccines. Additionally, 7% of subjects (*N* = 7) received a first dose of adenovirus viral vector vaccine followed by mRNA formulations. In our cohort, 36 patients (33%) had previous infection diagnosed by RT-PCR, and two of them experienced a second infection (2%).

In particular, of those subjects, 43% (*N* = 15) were not-fully vaccinated (less than 3 doses) and 57% fully vaccinated (3 or more doses).

### Neutralizing antibody titers against ancestral and Omicron BA.1 variants

Neutralizing antibody titers were assessed at a median time of 135 days (range, 15–504 days) since the most recent vaccine dose or SARS-CoV-2 infection. In 9% (*N* = 9) or 21% (*N* = 20) of cases, the time from the last vaccine dose or SARS-CoV-2 infection to antibody evaluation was less than 30 days or more than 180 days, respectively (Table 1). A measurable neutralizing antibody response was observed in 71% and 82% cases for ancestral and Omicron BA.1 variant, respectively (Table 2). In 6 patients (6%), neutralizing antibody titer was not measurable for either variant. Specifically, anti-wt antibody titers of 1:10 were observed in 19% of subjects (MM, *N* = 4; lymphomas, *N* = 7; MDS, *N* = 2; acute/chronic leukemias, *N* = 6), titers of 1:40 in 27% of cases (MM, *N* = 10; lymphomas, *N* = 5; MDS, *N* = 6; acute leukemias, *N* = 4; others, *N* = 1), titers of 1:160 in 14% of patients (MM, *N* = 1; lymphomas, *N* = 2; MDS, *N* = 2; acute/chronic leukemias, *N* = 4; and others, *N* = 1), and titers of 1:640 in 10% of subjects (MM, *N* = 3; MDS, *N* = 2; acute/chronic leukemias, *N* = 4). In 20% of cases, neutralizing antibody titers were < 1:10 (MM, *N* = 2; lymphomas, *N* = 10; MDS, *N* = 2; acute/chronic leukemias, *N* = 6; and idiopathic myelofibrosis, *N* = 1). These titers were similar to those observed in a group of healthy controls from another our parallel study (unpublished data), in which a measurable neutralizing antibody response was observed in 95% of subjects. In details, in healthy controls, titers were 1:10 in 15% of cases, 1:40 in 40%, 1:160 in 25%, and 1:640 in 15% of subjects. No differences were documented in neutralizing antibody responses between hematological patients and healthy subjects (*P* = 0.2696).

**Table 2:** Serological outcomes

Characteristics	N = 106
Anti-SARS-CoV-2 neutralizing antibody response, * n* (%)	
*Wild type virus*	75 (71)
*Omicron BA.1*	87 (82)
*Failed culture*	6 (6)
Anti-ancestral neutralizing antibody titer, * n* (%)	
< 1:10	25 (24)
1:10	20 (19)
1:40	29 (27)
1:160	15 (14)
1:640	11 (10)
Failed culture	6 (6)
Anti-Omicron BA.1 neutralizing antibody titer, * n* (%)	
< 1:10	13 (12)
1:10	34 (32)
1:40	26 (25)
1:160	25 (24)
1:640	2 (2)

Titers of anti-BA.1 antibodies were 1:10 in 32% of cases (MM, *N* = 8; lymphomas, *N* = 8; MDS, *N* = 6; acute/chronic leukemias, *N* = 8), 1:40 in 25% of subjects (MM, *N* = 5; lymphomas, *N* = 5; MDS, *N* = 5; acute leukemias, *N* = 4; and idiopathic myelofibrosis, *N* = 1), 1:160 in 24% of patients (MM, *N* = 5; lymphomas, *N* = 6; MDS, *N* = 2; acute/chronic leukemias, *N* = 7; others, *N* = 2), and 1:640 only in one subject with acute lymphoblastic leukemia. In 11% of cases, neutralizing antibody titers were < 1:10 (MM, *N* = 1; lymphomas, *N* = 6; MDS, *N* = 1; acute/chronic leukemias, *N* = 4). Proportions of patients who had neutralizing antibodies against the ancestral virus were similar to those who developed neutralizing antibodies against BA.1 variant, regardless the type of underlying hematological condition (all *P* > 0.05; Chi square test performed), although sample size of compared groups was small. Patients who have received vaccine or have been infected within 30 or 180 days before antibody level measurement still displayed an adequate response against both ancestral virus (78% and 81%, respectively) and BA.1 variant (100% and 95%, respectively). This response was similar to that observed in those who have received vaccine or got infected between 30 and 180 days before antibody level measurement (all *P* > 0.05). Moreover, patients were also stratified by number of vaccine doses, and antibody response against wt virus was slightly inferior in subjects who received ≤ 2 doses compared to fully vaccinated patients (*N* = 18 and *N* = 60, respectively; 61% vs 79%, not-fully vaccinated vs fully vaccinated subjects; *P* = 0.075). Conversely, antibody response against Omicron BA.1 variant was similar in both fully and not-fully vaccinated subjects (*N* = 65 and *N* = 23, respectively; 85% vs 78%, fully vaccinated vs not-fully vaccinated subjects; *P* = 0.48).

### Factors influencing neutralizing antibody titers

**Fig. 1:**
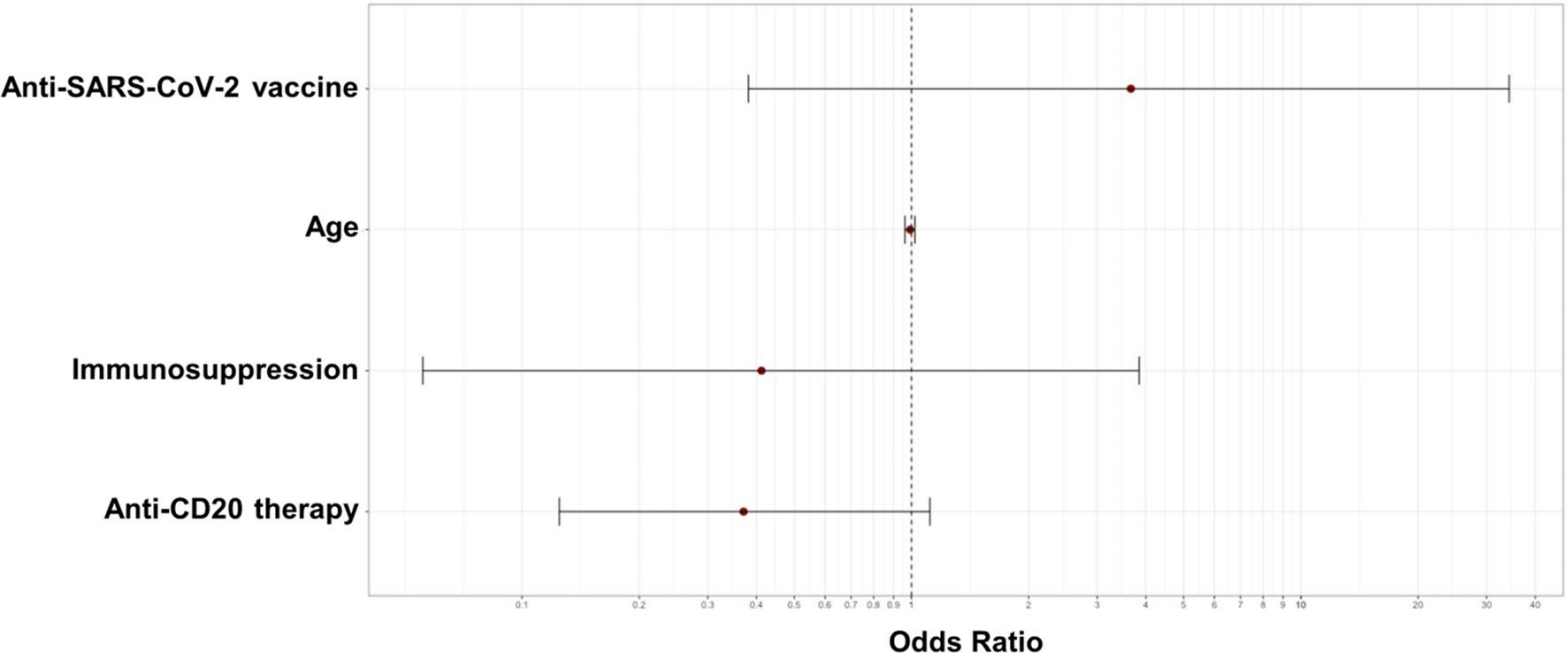
Forest plot showing the results of multivariate logistic regression analysis for the presence of neutralizing antibody response against ancestral virus. The *x*-axis represents the Odds ratio with the reference line (dashed), Odds ratios (circle) and 95% Confidential Interval (whiskers)

Next, we investigated the influence of several clinical features, such as type of treatment and monoclonal antibodies used, on neutralizing antibody production. In our cohort, patients treated with anti-CD20 monoclonal antibodies showed a significant decreased antibody response against the Omicron BA.1 variant compared to subjects not treated with anti-CD20 agents (73% vs 91%, with anti-CD20 vs without anti-CD20-based treatment; *P* = 0.02). However, no differences were observed for antibody response against the ancestral virus (64% vs 78%; *P* = 0.16). Both univariate (Table 3; dependent variable, presence of neutralizing antibody response against ancestral virus) and multivariate logistic regression did not document significant associations with an adequate anti-wt antibody response (Table 4; dependent variable, presence of neutralizing antibody response against ancestral virus) (Fig. 1). In contrast, a univariate logistic regression on evaluable anti-Omicron BA.1 antibody response described a significant direct association with anti-SARS-CoV-2 vaccines (odds ratio [OR] 7.2; 95% confidential interval [CI] 1.10–57.00; *P* = 0.05), while an inverse trend was observed for anti-CD20 monoclonal antibody therapy administered in the last 12 months (OR 0.26; 95%CI 0.07–0.88; *P* = 0.03) and diagnosis of NHL (OR 0.29; 95%CI 0.04–0.95; *P* = 0.04) (Table 5; dependent variable, presence of neutralizing antibody response against Omicron BA.1 variant). Additionally, no associations were found with the time of most recent vaccination or infection to antibody titer assessment (OR 3.25; 95%CI 0.43–29.6; *P* = 0.23).

**Table 3:** Univariate logistic regression for wild type virus

Variable	Sample size	OR	95% CI	*P* value
Sex (male)	65	1.31	0.52–3.30	0.55
Age (years)	–	1	0.97–1.02	0.97
Anti-SARS-CoV-2 vaccine, yes	94	3.1	0.41–23.5	0.26
Prior natural SARS-CoV-2 infection	36	0.73	0.28–1.93	0.53
Anti-CD20 therapy	22	0.48	0.17–1.35	0.16
Anti-CD38 therapy	14	2.10	0.45–10.5	0.32
Immunosuppressive agents	6	0.47	0.07–3.04	0.43
Azacytidine	23	0.92	0.32–2.69	0.89
High-dose chemotherapy	26	0.57	0.21–1.57	0.28
Monovalent mRNA vaccination, yes	82	1.2	0.21–6.67	0.83
> 30 days from last exposure, yes	97	1.05	0.2–5.5	0.95
> 60 days from last exposure, yes	24	1.18	0.37–3.72	0.77
> 90 days from last exposure, yes	36	1.07	0.39–2.98	0.88
> 180 days from last exposure, yes	21	1.3	0.39–4.63	0.62
Multiple myeloma, yes	24	2.66	0.72–9.89	0.14
Myelodysplastic syndrome, yes	14	5.01	0.62–40.6	0.13
Acute leukemias, yes	22	0.66	0.22–1.99	0.46
Non-Hodgkin Lymphoma, yes	22	0.49	0.15–1.39	0.17
Hodgkin Lymphoma, yes	7	0.39	0.08–1.90	0.24
Chronic myeloid leukemia, yes	4	Not evaluable	–	0.99
Chronic lymphocytic leukemia, yes	3	0.65	0.06–7.58	0.73
Allogeneic stem cell transplant, yes	7	0.48	0.08–3.05	0.44
Total exposures (vaccines + infections)	–	1.20	0.75–1.90	0.43

**Table 4:** Multivariate logistic regression for wild type virus

Variable	OR	95% CI	*P* value
Age (years)	0.99	0.96–1.02	0.60
Anti-CD20 therapy	0.37	0.12–1.09	0.07
Immunosuppressive agents	0.41	0.05–3.09	0.38
Anti-SARS-CoV-2 vaccine, yes	3.66	0.44–30.1	0.22

**Table 5:** Univariate logistic regression for Omicron BA.1 variant

Variable	Sample size	OR	95% CI	*P* value
Sex (male)	65	2.3	0.60–9.10	0.21
Age (years)	-	0.9	0.96–1.03	0.92
Anti-SARS-CoV-2 vaccine, yes	94	7.2	1.10–57.00	0.05
Prior natural SARS-CoV-2 infection	36	0.91	0.26–3.1	0.88
Anti-CD20 therapy	22	0.26	0.07–0.88	0.03
Anti-CD38 therapy	14	2.10	0.25–17.60	0.45
Immunosuppressive agents	6	0.57	0.06–5.61	0.63
Azacytidine	23	1.75	0.35–8.53	0.48
High-dose chemotherapy	26	0.67	0.18–2.41	0.54
Monovalent mRNA vaccination, yes	82	1.16	0.12–10.72	0.89
> 30 days from last exposure, yes	97	Not evaluable	–	0.99
> 60 days from last exposure, yes	24	0.62	0.16–2.36	0.48
> 90 days from last exposure, yes	36	0.54	0.15–1.94	0.34
> 180 days since last exposure, yes	21	3.25	0.43–29.6	0.23
Multiple myeloma, yes	24	4.06	0.50–33.1	0.19
Myelodysplastic syndrome, yes	14	2.11	0.25–17.6	0.49
Acute leukemias, yes	22	1.34	0.27–6.60	0.72
Non-Hodgkin lymphoma, yes	22	0.29	0.04–0.95	0.04
Hodgkin lymphoma, yes	7	0.92	0.10–8.14	0.9
Chronic myeloid leukemia, yes	4	Not evaluable	–	0.99
Chronic lymphocytic leukemia, yes	3	0.28	0.02–3.36	0.31
Allogeneic stem cell transplant, yes	7	0.58	0.06–5.61	0.64
Total exposures (vaccines + infections)	–	1.12	0.62–2.1	0.70

Previous SARS-CoV-2 infection occurred in 46% of patients who did not developed neutralizing antibodies against ancestral virus compared to 33% of subjects who had an adequate response (*P* = 0.36), as well 44% without adequate antibody response and 32% with appropriate neutralizing antibody titers against BA.1 variants (*P* = 0.28). Moreover, in the under-vaccinated population (≤ 2 doses of vaccine; *N* = 15), previous SARS-CoV-2 infection occurred in 57% of patients who did not developed neutralizing antibodies against ancestral virus compared to 75% of subjects with an adequate response (*P* = 0.46), and in 50% of patients without adequate response against BA.1 variant compared to 69% of subjects with appropriate antibody titers (*P* = 0.59).

**Fig. 2:**
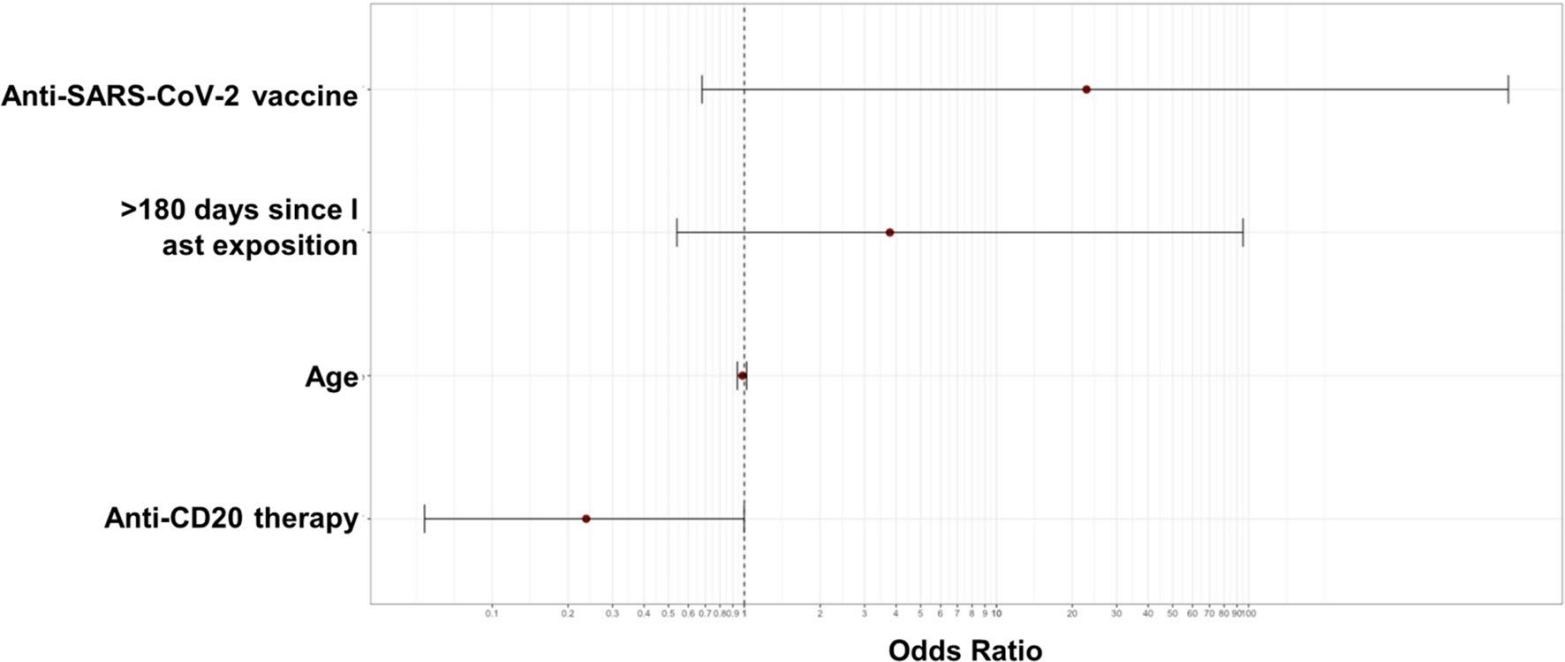
Forest plot showing the results of multivariate logistic regression analysis for the presence of neutralizing antibody response against the Omicron BA.1 variant. The *x*-axis represents the Odds ratio with the reference line (dashed), Odds ratios (circle) and 95% Confidential Interval (whiskers)

By multivariate logistic regression, only anti-CD20 monoclonal antibody therapy within prior 12 months retained inverse statistical significance (OR 0.23; 95%CI 0.05–0.98; *P* = 0.04) (Table 6; dependent variable, presence of neutralizing antibody response against Omicron BA.1 variant) (Fig. 2). In Table 7, we summarized clinical and serological characteristics of multiple myeloma patients given the unexpectedly high antibody titer in this cohort.

**Table 6:** Multivariate logistic regression for Omicron BA.1 variant

Variable	OR	95% CI	*P* value
Age (years)	0.98	0.94–1.02	0.43
Anti-CD20 therapy	0.23	0.05–0.98	0.04
> 180 days since last exposure, yes	3.8	0.35–39.9	0.26
Anti-SARS-CoV-2 vaccine, yes	22.77	0.85–609	0.06

**Table 7:** Focus on multiple myeloma patients

Characteristics	*N* = 24
*Multiple myeloma regimens, n* (%)	
Daratumumab-bortezomib-thalidomide-dexamethasone	8 (33)
Daratumumab-lenalidomide-dexamethasone	5 (21)
Carfilzomib-lenalidomide-dexamethasone	3 (12)
Isatuximab-carfilzomib-dexamethasone	1 (4)
Others	7 (30)
*Anti-ancestral neutralizing antibody response*, * n* (%)	
Daratumumab-bortezomib-thalidomide-dexamethasone	7/8 (88)
Daratumumab-lenalidomide-dexamethasone	4/5 (80)
Carfilzomib-lenalidomide-dexamethasone	3/3 (100)
Isatuximab-carfilzomib-dexamethasone	1/1 (100)
Others	5/7 (71)
*Anti-Omicron BA.1 neutralizing antibody response*, * n* (%)	
Daratumumab-bortezomib-thalidomide-dexamethasone	7/8 (88)
Daratumumab-lenalidomide-dexamethasone	5/5 (100)
Carfilzomib-lenalidomide-dexamethasone	3/3 (100)
Isatuximab-carfilzomib-dexamethasone	1/1 (100)
Others	6/7 (86)

## Discussion

COVID-19 is a pandemic disease characterized by severe respiratory symptoms, often requiring hospitalizations and intensive care, and also non-respiratory syndromes, such as cytokine release storm and thrombotic events [26–29]. Approval of effective anti-SARS-CoV-2 vaccines has dramatically reduced the incidence of severe COVID-19 and related deaths [30]. However, cancer and immunosuppressed patients develop a poor antibody response following vaccination and/or SARS-CoV-2 infection [6]. Additionally, numerous novel SARS-CoV-2 variants are rapidly identified, arising concerns regarding the ability of antibodies against the ancestral wt virus to neutralize new circulating variants, including Omicron BA.1 and its sub-lineages. In this retrospective real-life study, we reported anti-SARS-CoV-2 neutralizing antibody serostatus using a live virus assay in patients with different hematologic malignancies, and clinical features influencing neutralizing antibody development. Before vaccine approvals, neutralizing antibody rates range from very low (about 30%) in hospitalized COVID-19 patients with severe disease to 71.9%, with high inter-trial variability [31–36]. In post-vaccination era, rates of neutralizing antibody activity are between 37 and 66% after one or two doses in hematological patients and neutralizing antibody response rates are 27–50% after two doses [37]. Moreover, only a small proportion of COVID-19 patients or vaccinated subjects (26.7% and 38.2%, respectively) achieve an adequate titer against Omicron variants [38]. In our cohort, overall rates of neutralizing antibody activity were 71% and 82% for wt virus and Omicron BA.1 variant, respectively. These rates were higher than those previously reported, likely because the majority of our patients (73%) have received three or more mRNA-based or mixed vaccines. In addition, prior SARS-CoV-2 infection was not associated with higher neutralizing antibody titers against both wt virus and Omicron BA.1 variant, confirming clinical efficacy of anti-SARS-CoV-2 vaccines in producing adequate and prolonged antibody responses, as observed in univariate and multivariate analysis. However, most of previous SARS-CoV-2 infections occurred in patients vaccinated with ≥ 2 doses, likely because of the high prevalence of these subjects in our cohort. Therefore, SARS-CoV-2 infection occurred regardless the number of vaccine doses.

In our cohort, detectable neutralizing antibodies were observed even after more than 180 days since the last vaccine dose or SARS-CoV-2 infection. Indeed, in both multivariate models, anti-COVID-19 vaccination highly increased the probability of achieving an adequate neutralizing anti-SARS-CoV-2 antibody titer, demonstrating that natural infection alone is not sufficient in providing effective COVID-19 protection. Several risk factors have been associated with severe COVID-19 [7], such as cancers due to associated immunosuppression status and chemotherapy-related leukopenia with increased risk of infections [39–42]. Chronic lymphocytic leukemia patients display the lowest seropositivity rate (51%), even after receiving two doses of mRNA-based vaccine (43%) [37, 43–45], as well as old MM patients (42.4–71%) [46]. Conversely, patients with acute (93%) or chronic myeloid leukemia (87.5%) show high seropositivity rates even after just one dose of mRNA-based vaccine [37, 47]. In our cohort, lymphoma patients exhibited the lowest seropositivity rate, regardless of the number of doses for both wt (58.3%) and Omicron BA.1 variant (76%). On the other hand, multiple myeloma patients showed the highest seropositivity rate for both wt virus (90%) and its variant (95%). Our higher seropositivity rate in multiple myeloma patients compared to previous reports may be due to the larger number of fully vaccinated (more than two doses received) subjects in our cohort. Although our chronic lymphocytic leukemia patients displayed an adequate neutralizing antibody titer against both ancestral virus and its variant, the number of subjects was limited and we could not draw any definitive conclusions. Conversely, our lymphoma patients had a lower seropositivity rate against wt virus and its variant, likely due to the immunosuppressive effects of anti-CD20 monoclonal antibody therapies administered in the prior 12 months before vaccination.

Low antibody responses and anti-CD20 targeted therapies have been reported after a single mRNA-based vaccine dose in patients with different hematological malignancies [48–52]. However, these studies only evaluated anti-nucleocapsid and/or anti-spike antibodies. In contrast, our study measured neutralizing antibodies, responsible for severe COVID-19 protection. Very low rates of seroconversion have been previously reported for multiple myeloma or chronic lymphocytic leukemia patients, likely because of an earlier evaluation after just one or two vaccine doses and not in fully vaccinated subjects, as in our study [45, 46]. Our study included a heterogeneous hematological cohort, regardless of cancer type, and found high seropositivity rates and antibody titers against Omicron BA.1 variant in fully vaccinated hematological patients. Moreover, only anti-CD20 monoclonal antibody treatment was significantly associated with lower seropositivity rates and neutralizing antibody titers, especially for anti-Omicron BA.1 responses. Our results add evidence to the protective role of current approved vaccines, as Omicron variants are predominant worldwide. Our real-life study has some limitations: (i) Only B-cell-dependent humoral responses were explored; (ii) additional clinical risk factors were not included, and the number of patients with certain hematological diseases (e.g., idiopathic myelofibrosis) was limited, as we reported a single-center real-life study enrolling consecutive patients over a short period of time; (iii) our study design was limited to a retrospective investigation, while a prospective observation of dynamic decay of neutralizing antibody titers over time and during treatments could have added additional information regarding type and duration of humoral immune responses against SARS-CoV-2; (iv) other variants (e.g., XBB) were not tested, as those are closely related to Omicron and BA.1; and (v) likely asymptomatic or paucisymptomatic infections including those induced by Omicron BA.1 variant were more frequent of those diagnosed, explaining why the rate of anti-BA.1 antibodies was higher than anti-ancestral virus antibodies.

A strength of our study is the use of a live virus neutralization assay as a serological readout, as most studies use pseudoneutralization assays that serve as excellent surrogates while not fully measure the immunity provided by vaccination. This aspect of our work mirrors the “real-life” implications of neutralizing antibody production.

In conclusions, we documented a successful and sustained neutralizing antibody response against the ancestral virus and its Omicron BA.1 variant, even in a cancer-related immunosuppressed population. However, subjects diagnosed with B-cell lymphoproliferative disorders could have a lower immunization rate, likely because of undergoing anti-CD20 treatments. Therefore, full vaccination and conservation of protective measures, such as wearing masks indoor and in crowded places, should be always proposed to frail hematological patients to reduce infective risk to known pathogens.

## Data Availability

Data are available upon request by the authors.

## Declarations

### Statements

The authors declare that the material is original, has not been published before nor is under consideration in any journal.
